# Supplementary material for: Programmable catalysis by support polarization: elucidating and breaking scaling relations
Source: Nat Commun. 2023 Nov 28;14:7795. doi: 10.1038/s41467-023-43641-0 (PMC10684597; doi:10.1038/s41467-023-43641-0)
Supplement: Supplementary file 1 — Supplementary Information [file 41467_2023_43641_MOESM1_ESM.pdf]

Supplementary Information

Programmable Catalysis by Support Polarization: Elucidating and Breaking the Scaling Relations

Jung et al.

Supplementary Note 1. Constrained-forces calculations

Derivation in this section follows those of Stengel et al.[1] and Fu et al.[2] Response of crystalline materials to a finite electric field is important for various engineering applications, but calculating it requires formally defining bulk polarization in periodic solids, which was not done until 1990s, when the Berry phase formalism was introduced[3]. Later, following developments including the introduction of the equivalent Wannier charge-center formula for polarization, a first-principles approach of studying insulators under homogeneous electric field was developed. This approach involves minimization of electric enthalpy H with field-polarized Bloch functions[4,5].

$$H(\boldsymbol{\nu}, \mathcal{E}) = E(\boldsymbol{\nu}) - \Omega P(\boldsymbol{\nu})\mathcal{E} \tag{1}$$

where $\boldsymbol{\nu}$ is ionic and electronic coordinates of the system. E, P and $\mathcal{E}$ represent Kohn-Sham energy, polarization and electric field, respectively. Vector quantities such as $\boldsymbol{\mathcal{E}}$, $\mathbf{P}$ are represented as scalar considering unidirectional context. Solving for $\boldsymbol{\nu}$ that minimizes H is referred to as fixed-$\mathcal{E}$ calculations.

Later it was suggested that displacement field, D, is a more relevant fundamental variable for finite field calculations[6]. A new functional, internal energy U

$$U(\boldsymbol{\nu}, D) = E(\boldsymbol{\nu}) + \frac{\Omega}{2\epsilon_0}[D - P(\boldsymbol{\nu})]^2 \tag{2}$$

was introduced, and it was shown that the minimization of H at fixed $\mathcal{E}$ and minimization of U at fixed D are essentially same problem:

$$\left.\frac{\partial H}{\partial \boldsymbol{\nu}}\right|_{\mathcal{E}} = \left.\frac{\partial U}{\partial \boldsymbol{\nu}}\right|_{D} \tag{3}$$

The fixed-field calculations for systems with insulator/metal interfaces requires consideration of additional complexities. First, the partially occupied states of the system is incompatible with the Berry phase calculation of polarization of the system[1]. Also, the depolarization field which arises from incomplete screening of the bound charges modifies the dielectric properties of the system[7]. Stengel and Spaldin further developed a method to calculate polarization and fixed-field responses[1], while also explaining the origin of depolarization fields using series capacitor model[8,9].

The method developed by Stengel and Spaldin however, has some limitations for the purpose of this research. It is computationally expensive, as it requires full calculation of hybrid Wannier functions[10] at each iterative step to determine the electric enthalpy or displacement field. This is demanding for computations describing surface chemistry that require multiple

adsorbates and surface coverages. Ideally, studying a polarized state at fixed field should be no slower than studying catalyst properties without field. Alternatively, the constrained-forces approach from Fu and Bellaiche[2] provides faster and more efficient computational capability for to vacuum-separated metal/insulator/metal heterostructure. Using equation (1) and imposing fixed-$\mathcal{E}$ (and equivalently at fixed-D) gives

$$\left.\frac{\partial H(\boldsymbol{\nu},\mathcal{E})}{\partial \boldsymbol{\nu}}\right|_{\mathcal{E}} = \frac{\partial E(\boldsymbol{\nu})}{\partial \boldsymbol{\nu}} - \Omega \frac{\partial P(\boldsymbol{\nu})}{\partial \boldsymbol{\nu}}\mathcal{E} \tag{4}$$

By separating $\boldsymbol{\nu}$ into ionic coordinates $\mathbf{R}$ and electronic coordinates $\boldsymbol{\mu}$ and minimizing H with respect to only $\boldsymbol{\mu}$, so that $H(\mathbf{R},\mathcal{E}) = \min_{\boldsymbol{\mu}} H(\mathbf{R},\boldsymbol{\mu},\mathcal{E})$, equation (4) can be re-written,

$$\left.\frac{\partial H(\mathbf{R},\mathcal{E})}{\partial R_i}\right|_{\mathcal{E}} = \frac{\partial E}{\partial R_i} - \Omega \frac{\partial P}{\partial R_i}\mathcal{E} \tag{5}$$

The first term on the right hand side is the Hellmann-Feynman forces, $-F$, and second term on the right side includes the Born effective charges (BECs), which are the derivative of polarization with displacement of atoms[11],

$$Z^*_{i\alpha\beta} = \frac{\Omega}{e}\frac{\partial P_\alpha}{\partial R_{i\beta}} \tag{6}$$

where i is the atomic index, α is the direction of polarization P_α, β is the direction of displacement $R_{i\beta}$, Ω is the volume and e is the elementary charge. Considering electric field in only the z direction, the direction perpendicular to the slab, at $\left.\frac{\partial H}{\partial \mathbf{R}}\right|_{\mathcal{E}} = 0$,

$$\frac{\partial E}{\partial R_{i,\alpha}} = \Omega \frac{\partial P}{\partial R_{i,\alpha}}\mathcal{E} \tag{7}$$

Combined with eq. 6,

$$-\frac{\partial E}{\partial R_{i,x}} = F_{i,x} = -eZ^*_{i,zx}\mathcal{E}_z \tag{8}$$
$$-\frac{\partial E}{\partial R_{i,y}} = F_{i,y} = -eZ^*_{i,zy}\mathcal{E}_z \tag{9}$$
$$-\frac{\partial E}{\partial R_{i,z}} = F_{i,z} = -eZ^*_{i,zz}\mathcal{E}_z \tag{10}$$

Without off-diagonal components if the BEC tensor, this simplifies to

$$-\frac{\partial E}{\partial R_i} = F_i = -eZ_i^* \mathcal{E} \tag{11}$$

which means that at the equilibrium crystal structure at fixed internal electronic perturbation, the Hellmann-Feynman forces on the ions are proportional to their Born effective charges. Search for the geometry of this condition can be implemented using existing multivariable optimization methods that utilizes the first derivatives, such as the variable metric (quasi-Newton) method or the conjugate gradient method. The saddle points are only accesible with the methods based on Newton's method. The patch file for custom version of VASP 5.4.4 can be found at following url: `https://doi.org/10.5281/zenodo.10045191`

Supplementary Figures

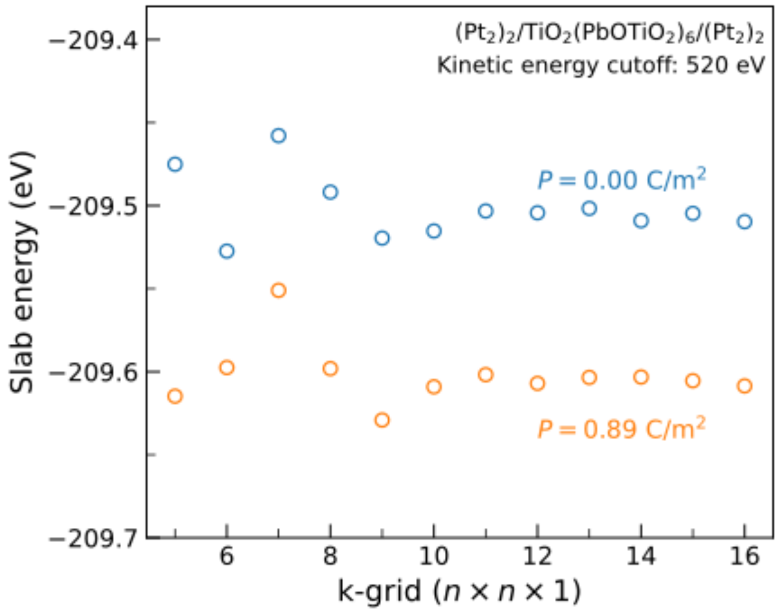

Supplementary Figure 1. $(Pt_2)_2/TiO_2(PbOTiO_2)_6/(Pt_2)_2$ structure energy convergence with **k**-grid. Two structures are at different values of polarization, 0.00 C/m^2 (metastable state) and 0.89 C/m^2 (spontaneous polarization). Kinetic energy cutoff is set to 520 eV.

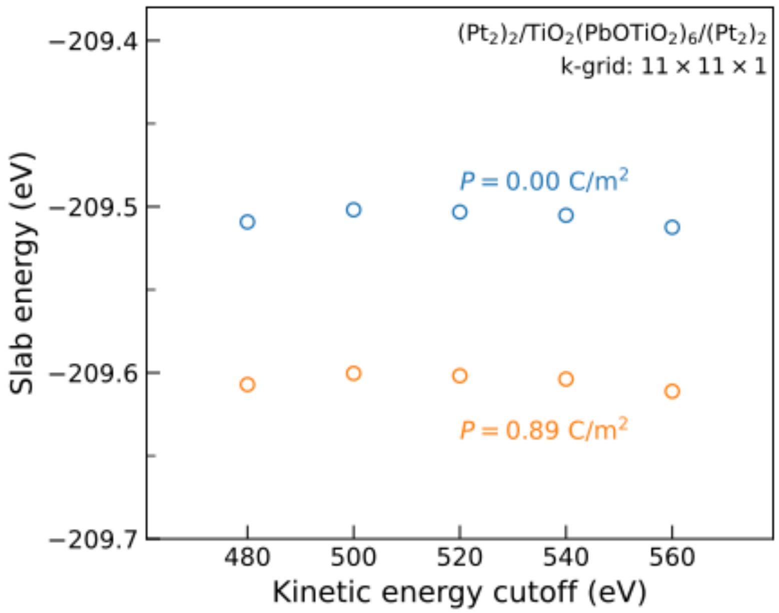

Supplementary Figure 2. $(Pt_2)_2/TiO_2(PbOTiO_2)_6/(Pt_2)_2$ structure energy convergence with kinetic energy cutoff. Two structures are at different values of polarization, 0.00 C/m^2 (metastable state) and 0.89 C/m^2 (spontaneous polarization). **k**-grid is set to $11 \times 11 \times 1$.

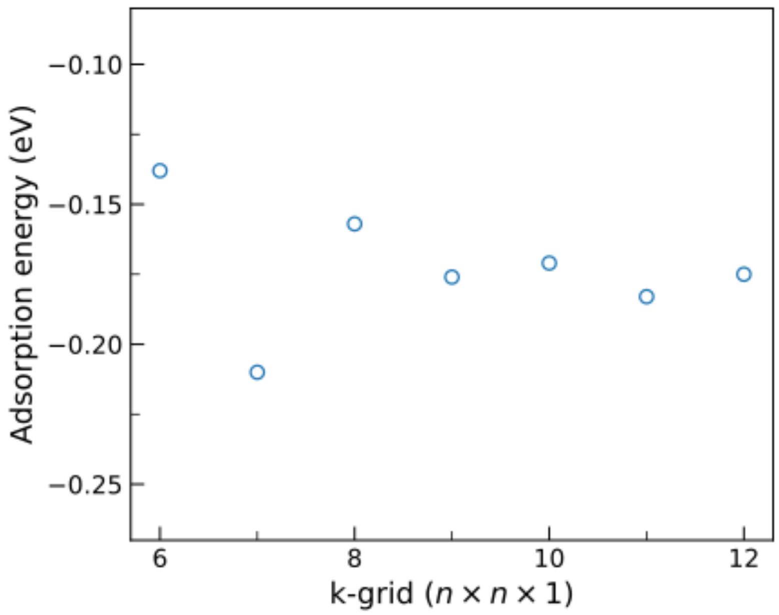

Supplementary Figure 3. Methanol adsorption energy on Pt (100) slab convergence with **k**-points.

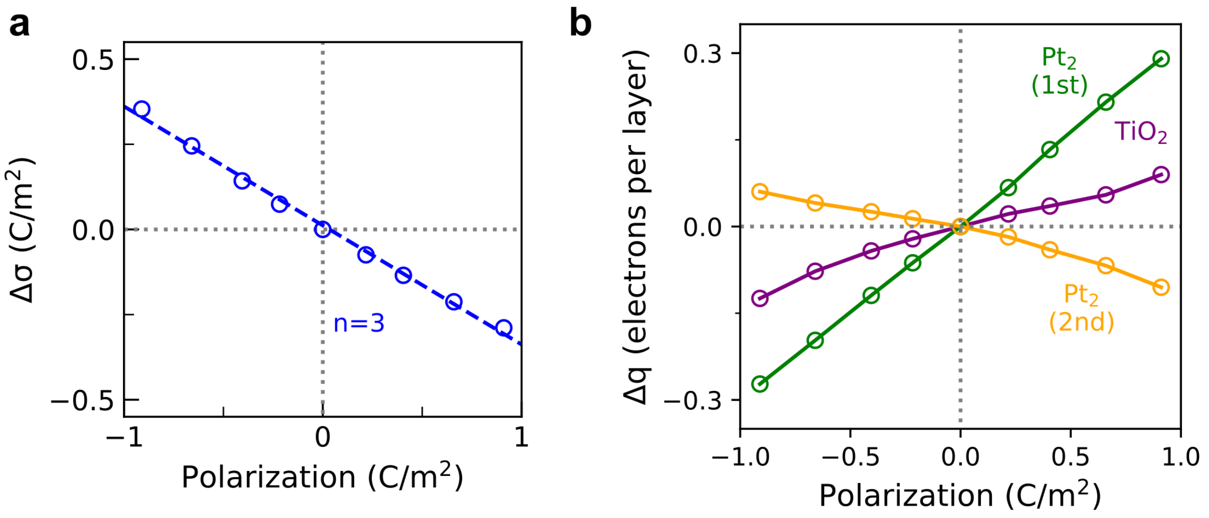

Supplementary Figure 4. DDEC6 charges response to support polarization $((Pt_2)_3/TiO_2(PbOTiO_2)_3/(Pt_2)_3)$. (a) Surface charge density difference at different polarization. (b) Bader charge difference per layer of interface TiO_2, interface 1st layer Pt_2 and 2nd layer Pt_2.

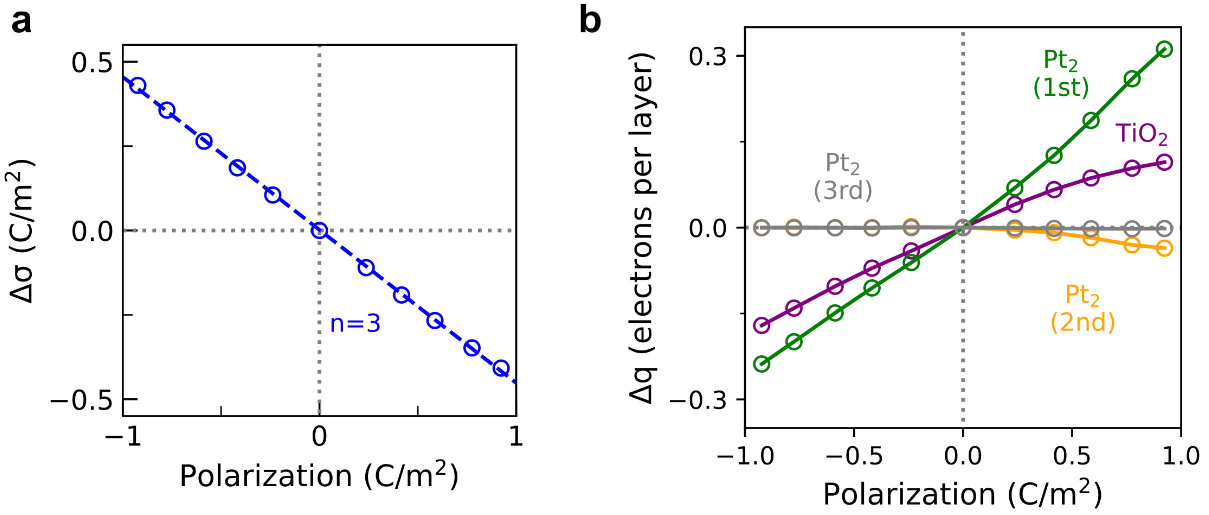

Supplementary Figure 5. Bader charges response to support polarization for 3 layer Platinum structure $((Pt_2)_3/TiO_2(PbOTiO_2)_3/(Pt_2)_3)$. (a) Surface charge density difference at different polarization. (b) Bader charge difference per layer of interface TiO_2, interface 1st layer Pt_2, 2nd layer Pt_2 and 3rd layer Pt_2.

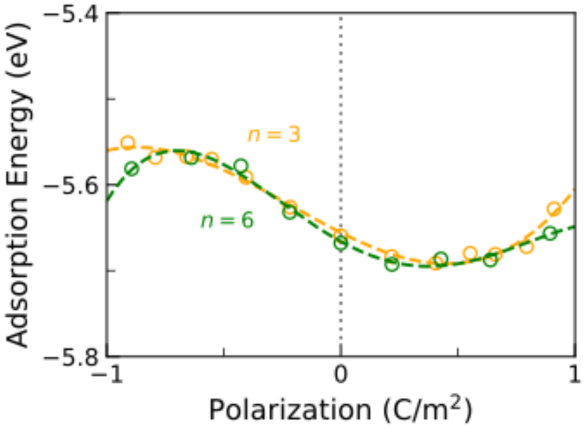

Supplementary Figure 6. C adsorption energies, top on A site of 2 layer Pt supported on 3.5 unit cells $PbTiO_3$ ($(Pt_2)_2/TiO_2(PbOTiO_2)_3/(Pt_2)_2$) structure and 6.5 unit cells $PbTiO_3$ ($(Pt_2)_2/TiO_2(PbOTiO_2)_6/(Pt_2)_2$) structure. Minor differences arise from work function changes from the adsorption, which affects the structures differently from their inherent electric field response. Dashed lines are fourth-order polynomial regression of the energies, for guidance.

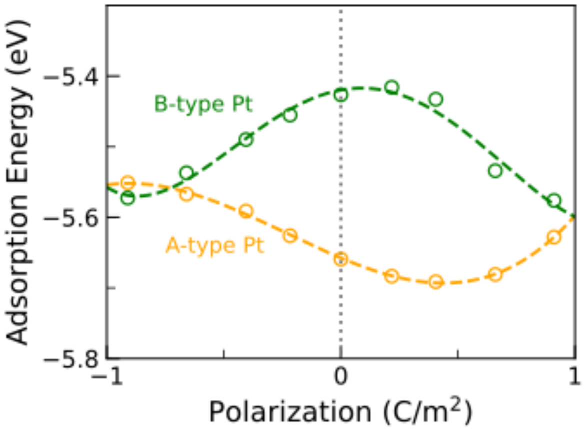

Supplementary Figure 7. C adsorption energies, on top A sites and and B sites of 2 layer Pt supported 3.5 unit cells $PbTiO_3$ ($(Pt_2)_2/TiO_2(PbOTiO_2)_3/(Pt_2)_2$) structure. Dashed lines are fourth-order polynomial regression of the energies, for guidance.

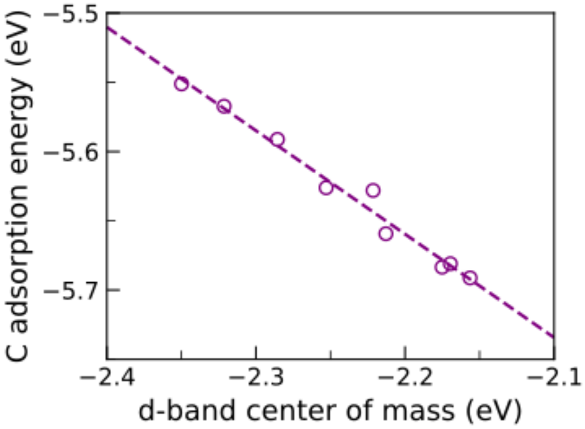

Supplementary Figure 8. The adsorption energies of C adatom on Pt(100) correlate linearly with the d-band center of mass of Pt atoms. Each data points are for different values of support polarization. While the d-band center of mass changes does not correlate linearly with all the adsorption energy changes for different adsorbates and adsorption sites, in the cases of minimum structural distortion, the d-band center of mass and adsorption energy changes show high correlation.

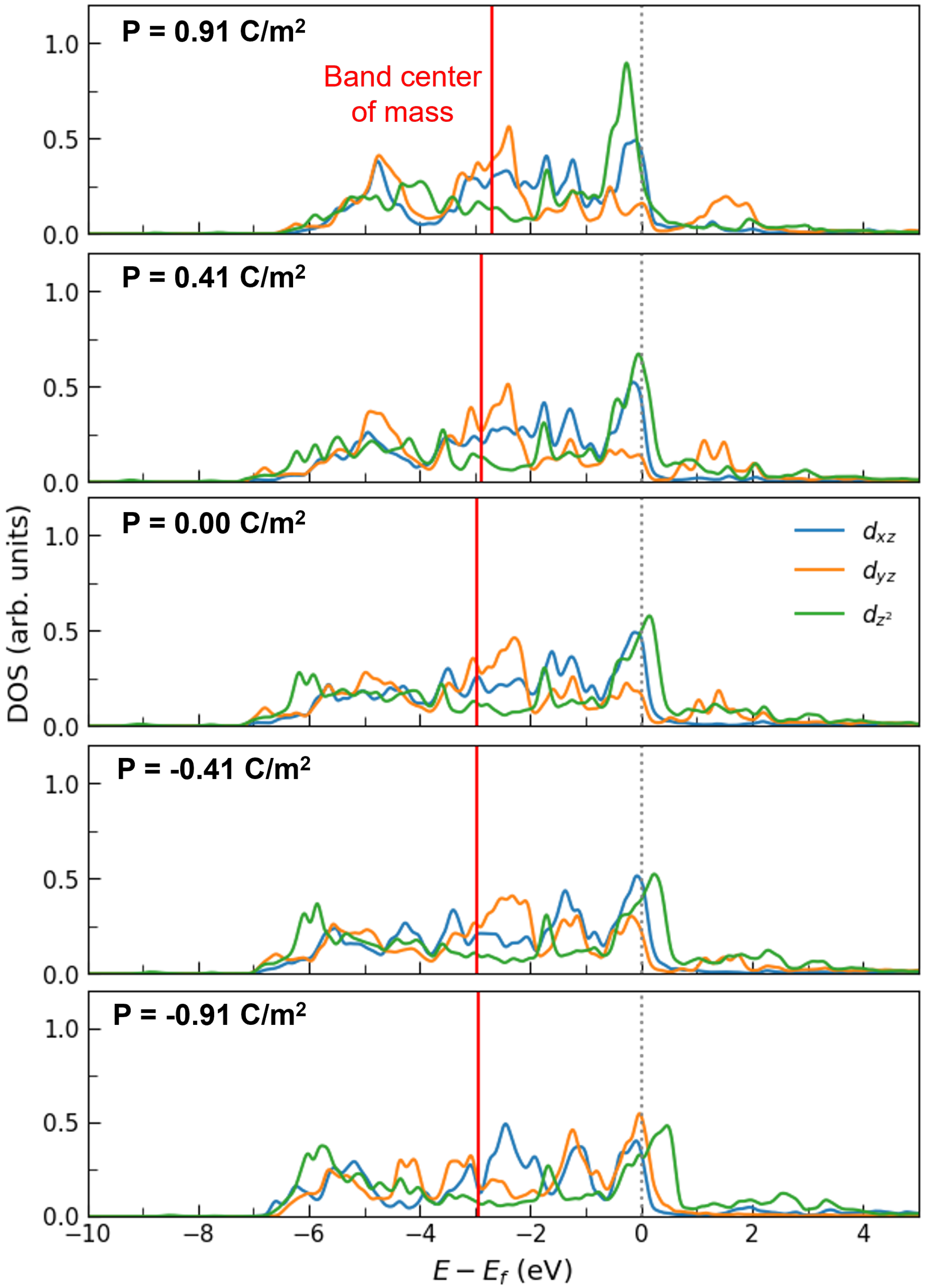

Supplementary Figure 9. Density of States of 1st layer Pt, in $(Pt_2)_2/TiO_2(PbOTiO_2)_3/(Pt_2)_2$. Dotted gray line represents the Fermi level, and the red line represents the *d*-band center of mass.

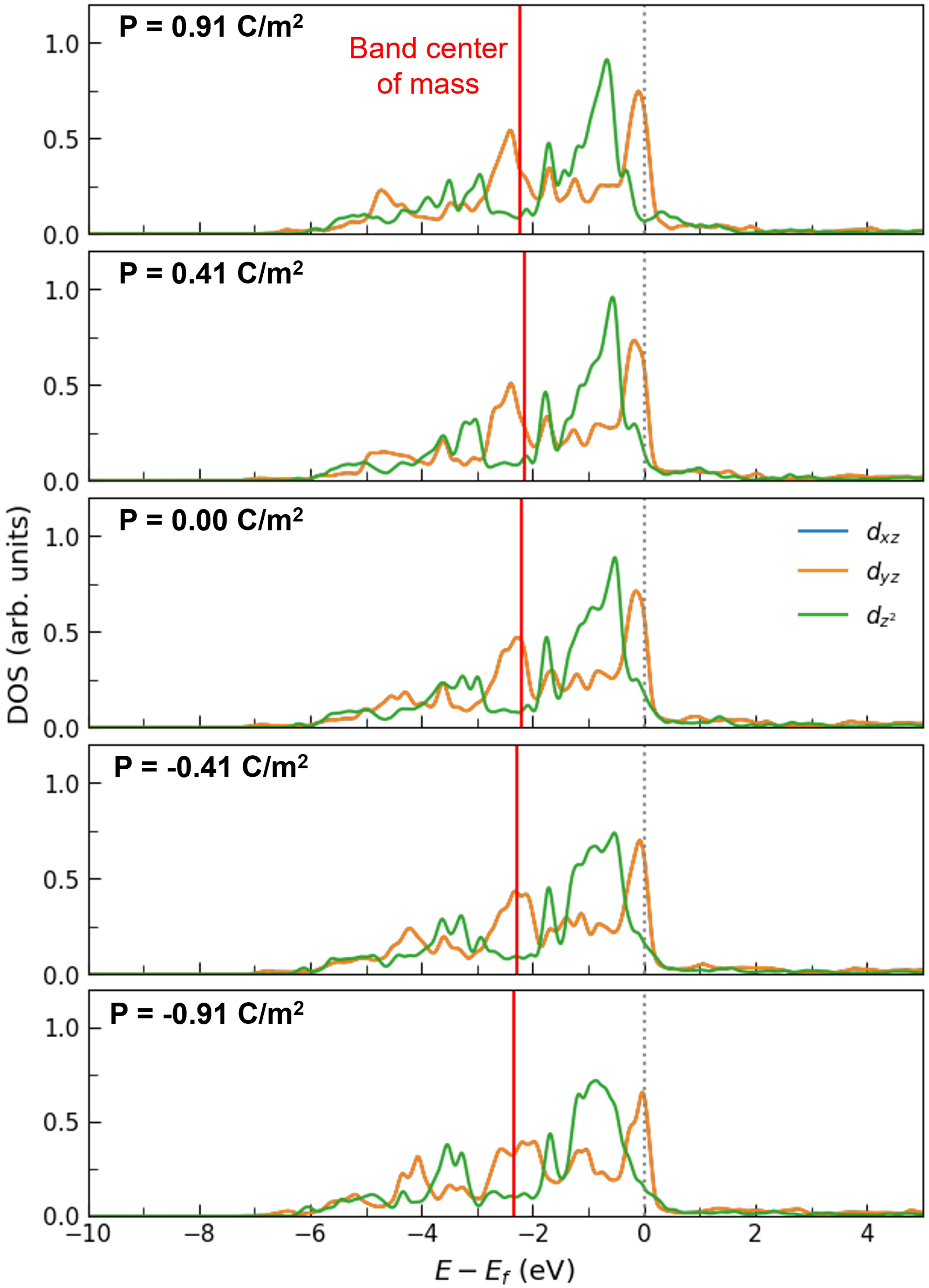

Supplementary Figure 10. Density of States of 2nd layer A-type Pt, in $(Pt_2)_2/TiO_2(PbOTiO_2)_3/(Pt_2)_2$. Dotted gray line represents the Fermi level, and the red line represents the d-band center of mass. d_{xz} and d_{xz} orbitals are equivalent for this Pt atom.

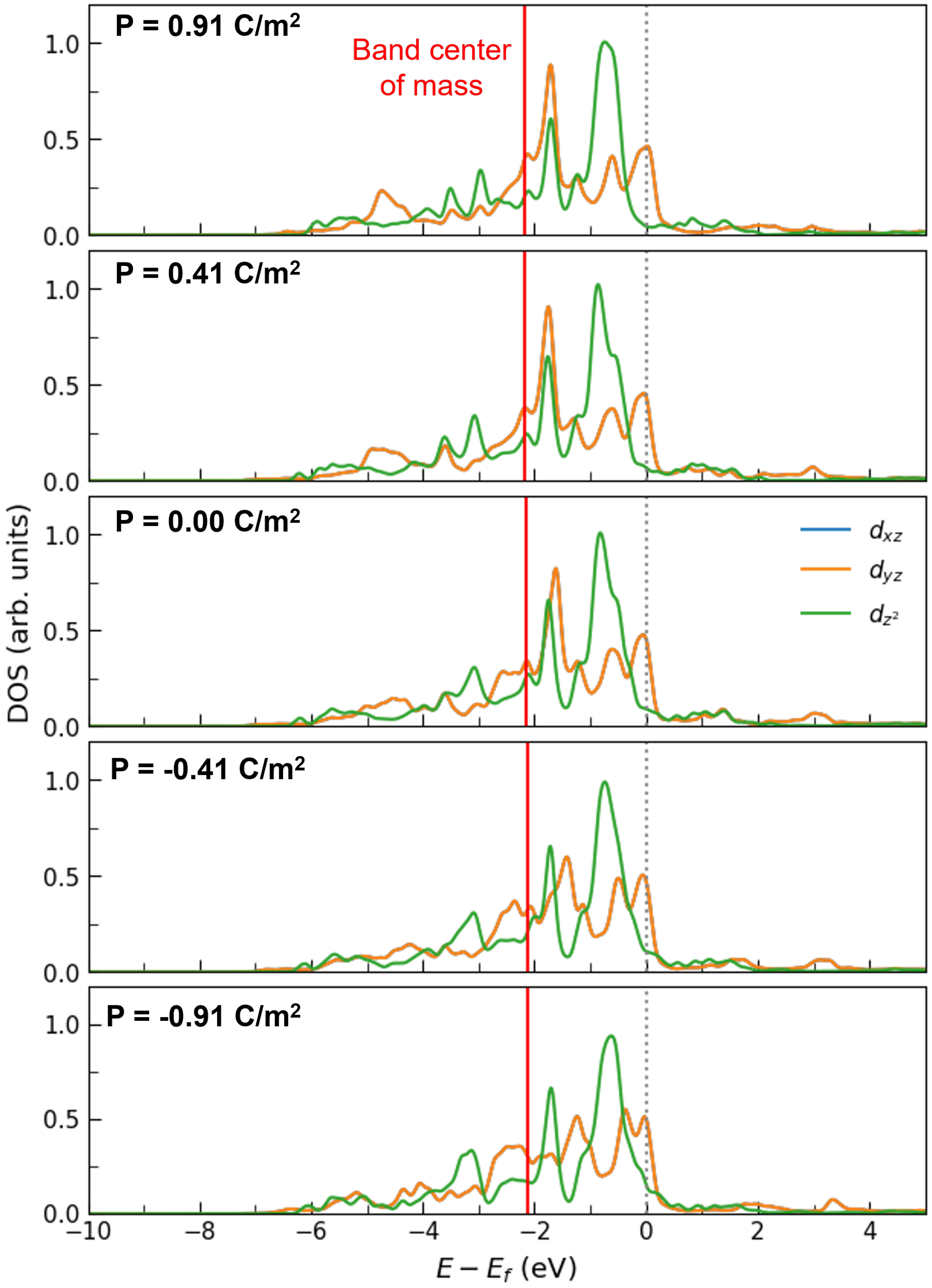

Supplementary Figure 11. Density of States of 2nd layer B-type Pt, in $(Pt_2)_2/TiO_2(PbOTiO_2)_3/(Pt_2)_2$. Dotted gray line represents the Fermi level, and the red line represents the d-band center of mass. d_{xz} and d_{xz} orbitals are equivalent for this Pt atom.

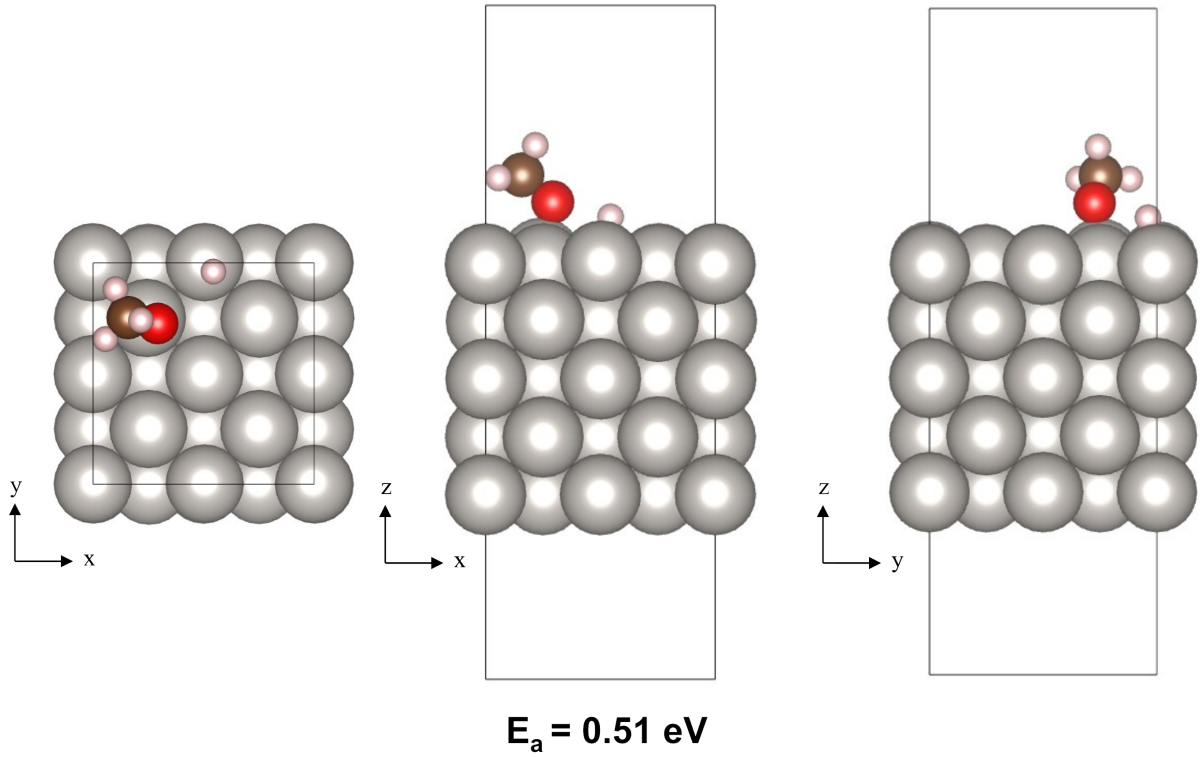

Supplementary Figure 12. Transition-state for minimum energy pathway of O-H bond dissociation on Pt (001) slab. Grey, brown, red, and pink atoms represent Pt, C, O, and H each.

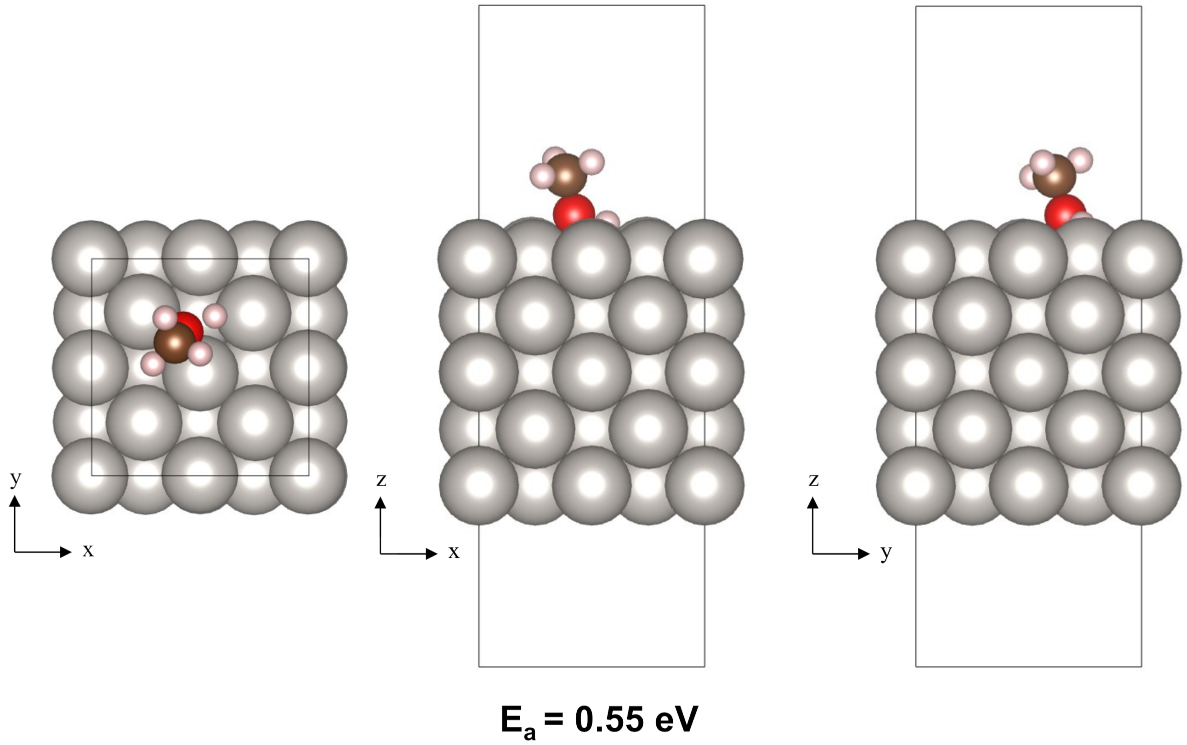

Supplementary Figure 13. Transition-state for an alternate energy pathway of O-H bond dissociation on Pt (001) slab. Grey, brown, red, and pink atoms represent Pt, C, O, and H each.

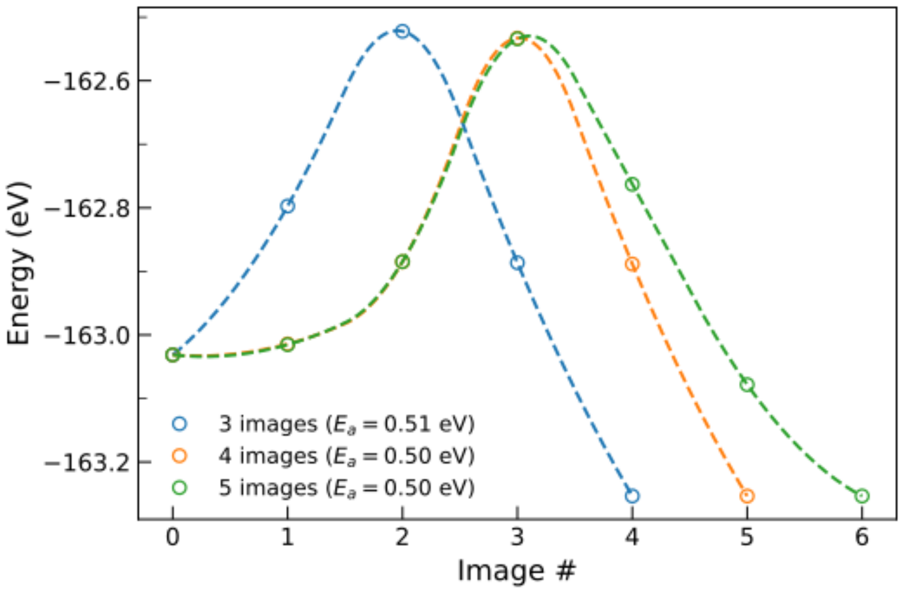

Supplementary Figure 14. Climbing image nudged elastic band minimum energy pathway convergence with different number of intermediate images. 3, 4, and 5 intermediate images between initial and final state results in same activation energy and transition state. Dashed lines are 2nd-degree spline interpolation for guidance.

Supplementary References

1. Stengel, M. & Spaldin, N. A. Ab initio theory of metal-insulator interfaces in a finite electric field. *Phys. Rev. B* **75,** 205121 (2007).
2. Fu, H. & Bellaiche, L. First-principles determination of electromechanical responses of solids under finite electric fields. *Phys. Rev. Lett.* **91,** 057601 (2003).
3. King-Smith, R. D. & Vanderbilt, D. Theory of polarization of crystalline solids. *Phys. Rev. B* **47,** 1651 (1993).
4. Nunes, R. W. & Gonze, X. Berry-phase treatment of the homogeneous electric field perturbation in insulators. *Phys. Rev. B* **63,** 155107 (2001).
5. Souza, I., Íniguez, J. & Vanderbilt, D. First-principles approach to insulators in finite electric fields. *Phys. Rev. Lett.* **89,** 117602 (2002).
6. Stengel, M., Spaldin, N. A. & Vanderbilt, D. Electric displacement as the fundamental variable in electronic-structure calculations. *Nat. Phys.* **5,** 304–308 (2009).
7. Wurfel, P. & Batra, I. Depolarization-field-induced instability in thin ferroelectric films —experiment and theory. *Phys. Rev. B* **8,** 5126 (1973).
8. Stengel, M. & Spaldin, N. A. Origin of the dielectric dead layer in nanoscale capacitors. *Nature* **443,** 679–682 (2006).
9. Stengel, M., Vanderbilt, D. & Spaldin, N. A. Enhancement of ferroelectricity at metal–oxide interfaces. *Nat. Mater.* **8,** 392–397 (2009).

10. Giustino, F. & Pasquarello, A. Theory of atomic-scale dielectric permittivity at insulator interfaces. *Phys. Rev. B* **71,** 144104 (2005).

11. Rabe, K. M. & Ghosez, P. in *Physics of Ferroelectrics: A Modern Perspective* 117–174 (Springer, 2007).
